# Metformin and endometrial cancer survival: a quantitative synthesis of observational studies

**DOI:** 10.18632/oncotarget.19830

**Published:** 2017-08-02

**Authors:** Jianfeng Guo, Kai Xu, Min An, Yingchao Zhao

**Affiliations:** ^1^ Department of Obstetrics and Gynecology, Union Hospital, Tongji Medical College, Huazhong University of Science and Technology, Wuhan 430022, China; ^2^ Department of Otolaryngology-Head and Neck Surgery, Tongji Hospital, Tongji Medical College, Huazhong University of Science and Technology, Wuhan 430030, China; ^3^ Cancer Center, Union Hospital, Tongji Medical College, Huazhong University of Science and Technology, Wuhan 430022, China; ^4^ ZhuJiang Hospital, Southern Medical University, Guangzhou 510515, China

**Keywords:** metformin, endometrial cancer, survival, quantitative synthesis, observational study

## Abstract

Metformin has been reported to have anticancer effect and can affect patient survival in several malignancies. However, the results are inconclusive for endometrial cancer. Hence, we conducted a systematic review and meta-analysis to investigate the prognostic role of metformin in patients with endometrial cancer. Studies were identified from Pubmed and Embase database through March 2017. Observational studies reporting hazard ratios (HRs) with 95% confidence intervals (CIs) for overall survival (OS) and progression-free survival (PFS) were selected. Data were abstracted and summarised using random-effects models. From 250 unique citations, we identified ten studies including 6242 patients with nine studies examining OS and five studies examining PFS. Meta-analysis demonstrated that metformin users had better OS (HR, 0.58; 95% CI, 0.45 to 0.76; *P* = 0.207, I^2^ = 26.6%) and PFS (HR, 0.61; 95% CI, 0.49 to 0.76; *P* =0.768, I^2^ = 0%) than non-users for endometrial cancer patients. Similar findings were observed using sensitivity analysis adjusted by trim and filled methods (HR, 0.47; 95% CI, 0.37 to 0.58) and subgroup analyses. Based on the current evidence, we find that metformin use is associated with better OS and PFS in patients with endometrial cancer. However, further large-scale prospective studies are needed to establish its validity.

## INTRODUCTION

Diabetes mellitus (DM) and cancer may share a variety of risk factors and pre-existing diabetes may increase the risk of death in patients with malignancy [1–6]. A growing number of studies have reported that patients with pre-existing diabetes have higher risk of developing endometrial cancer [7–12].

Metformin, a commonly prescribed glucose-lowering agent for the management of type 2 DM, is currently preferred as the first-line agent for patients with type 2 diabetes [13, 14]. Metformin has been reported to exert its antitumor effects through several mechanisms by activating LKB1/AMP-activated protein kinase (AMPK) pathway, inducing cell cycle arrest and/or apoptosis, inhibiting protein synthesis, activating the immune system and eradicating cancer stem cells [15].

Some epidemiological studies show a reduced risk of gynecological cancers such as endometrial cancer, ovarian cancer and breast cancer associated with metformin use in type 2 DM [16–18]. Mounting evidence suggests that metformin may affect the prognosis of several malignancies, including colorectal, lung, pancreatic, liver and endometrial cancer [19–22]. Nevertheless, evidence from current observational studies has not drawn definite conclusions whether metformin use significantly influences endometrial cancer patient survival. One study by Al Hilli et al. found metformin exposure was not associated with patients’ overall survival (OS) or progression-free survival (PFS) (hazard ratio [HR] 0.61; 95% confidence interval [CI] 0.30, 1.23 for OS and HR 1.06; 95% CI 0.34, 3.30 for PFS) [23]. However, another study by Pierce et al. revealed metformin use was significantly associated with improved OS (HR 0.49, 95% CI 0.34–0.71) and PFS (HR 0.60, 95% CI 0.43–0.84) [24]. Enhanced understanding and interpretation of the effect of metformin is helpful in determining adjuvant treatment strategies for endometrial cancer patients. In this study, we aimed to conduct a systematic review and meta-analysis for the evaluation of the relationship between metformin use and mortality in patients with endometrial cancer.

## RESULTS

### Study characteristics

**Figure 1:**
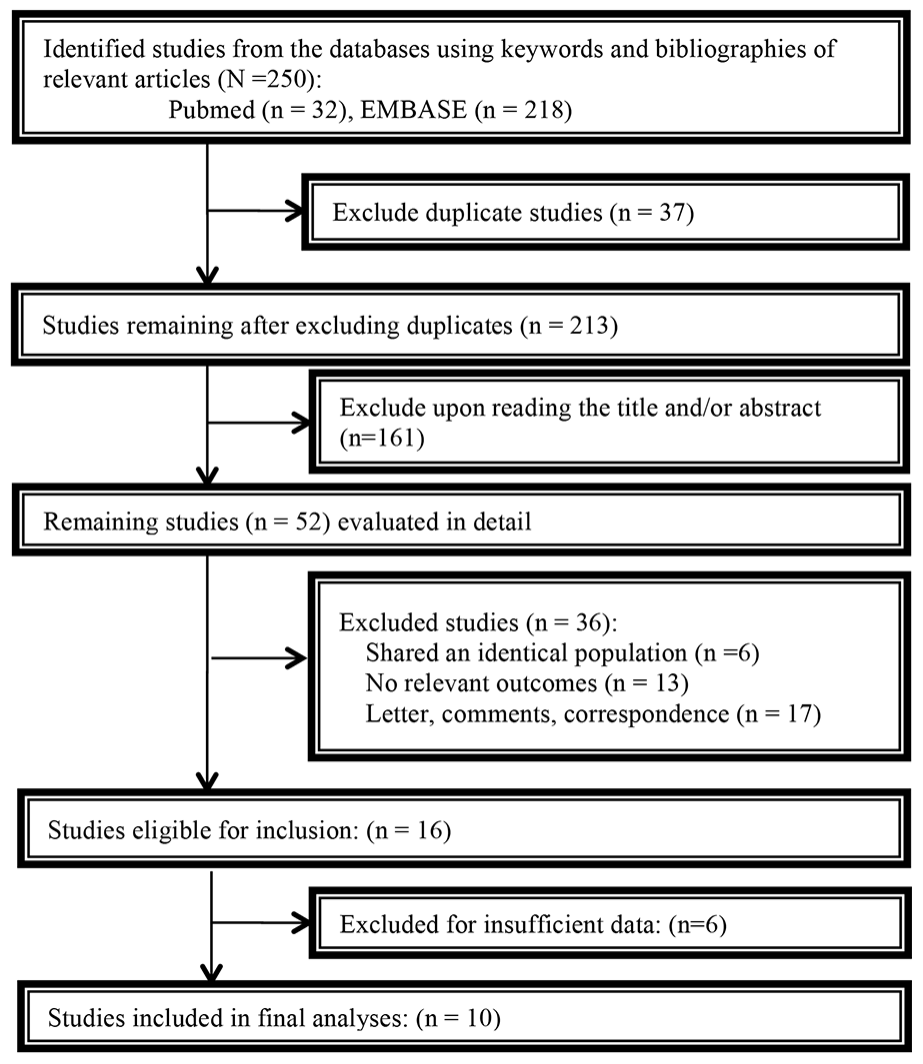
Flowchart of study selection

Figure 1 and Supplementary Table 1 outline the selection process of relevant studies. In summary, the search yielded a total of 250 unique citations, of which 52 met the criteria for further review. After full text screening, 36 were excluded because they shared an identical population (n=6), did not report relevant outcomes (n=13); were not original reports, such as letters, comments, correspondence (n=17) or did not include insufficient data for analyses (n=6). Finally, ten remaining studies were included for meta-analysis [23–32]. Baseline characteristics of the included studies are presented in Table 1. Ten studies including 6242 patients satisfying the inclusion criteria with nine studies examining OS and five studies examining PFS. The studies were mainly conducted in USA [23-28, 30, 31] except two [29, 32] and published between 2012 and 2016. The median sample size of the included studies was 465 (range, 107 to 1995), with a total of 924 metformin users and 5318 non-users. Nine of ten studies involved patients with stage I–IV disease and the other one involved stage I-III disease. Nine studies used multivariate analysis adjusted for covariates such as age, body mass index, stage, grade or treatment. Six of ten studies had a methodological quality score of more than seven (details in Supplementary Table 2).

**Table 1:** Baseline characteristics of included studies

Author (year)	Single or multicenter	Patients without DM	DM patients with MFM	DM patients without MFM	Inclusion period	Country of origin	Stage	Grade	Mean/median age (years)	Other treatment regimens	Follow-up Duration (months)	Reported endpoints	Adjusted variables
Seebacher (2016)	Single	NR	46	41	1995-2011	Australia	I-IV	G1-3	65.3	Operation, chemo- or radiotherapy	NR	OS	Age, stage, grade, histology, BMI
Ezewuiro (2016)	Multicenter	291	31	28	1992-2013	U.S.A	III-IV or relapse	NR	64	Chemotherapy	MFM:50;Non-MFM:54m; Non-DM:33m	OS	Study site, stage, age
Al Hilli (2016)	Single	1026	116	161	1999-2008	U.S.A	I-IV	G1-3	64.6	Operation, chemo- or radiotherapy	DM:52 Non-DM:62	OS, PFS	Age, stage, grade, histology, BMI, smoking status ,pulmonary dysfunction, radiation , hyperlipidemia
Freeman (2015)	Single	NR	32	153	1999-2013	U.S.A	NR	NR	61.5	NR	49	OS, DFS	NR
Lemanska (2015)	Single	39	30	38	2002-2010	Poland	I-III	G1-3	63	Operation, chemo- or radiotherapy	NR	OS	Age, stage, grade, radiation, operation, glucose level , BMI
Hahn (2014)	Single	348	51	46	2004-2010	U.S.A	I-IV	NR	NR	NR	NR	OS	NR
Ko (2014)	Multicenter	NR	200	163	2005-2010	U.S.A	I-IV	G1-3	MFM:62.2 ;Non-MFM:64.8	Chemo- or radiotherapy	33	OS, PFS	Age, race, BMI, stage, grade, histology , adjuvant treatment
Nevadunsky(2014)	Single	735	114	136	1999-2009	U.S.A	I-IV	G1-3	Non-DM:63.8;MFM:64.2;Non-MFM:64.1	Operation, chemo- or radiotherapy	40	OS	Age, stage, grade, histology, radiation ,hyperlipidemia
Pierce(2014)	Multicenter	1501	282	212	1997-2012	U.S.A	I-IV	NR	NR	NR	NR	OS, PFS	NR
Lin(2012)	Single	359	22	41	1991-2009	U.S.A	I-IV	NR	NR	Operation, chemo- or radiotherapy	NR	DFS	Stage, lymphovascular invasion, grade

BMI= body mass index; DFS=disease-free survival; DM=diabetes mellitus; MFM=metformin; NR=not reported; OS=overall survival; PFS=progression free survival; RC=retrospective cohort;

### Prognostic value of metformin use for endometrial cancer

**Figure 2:**
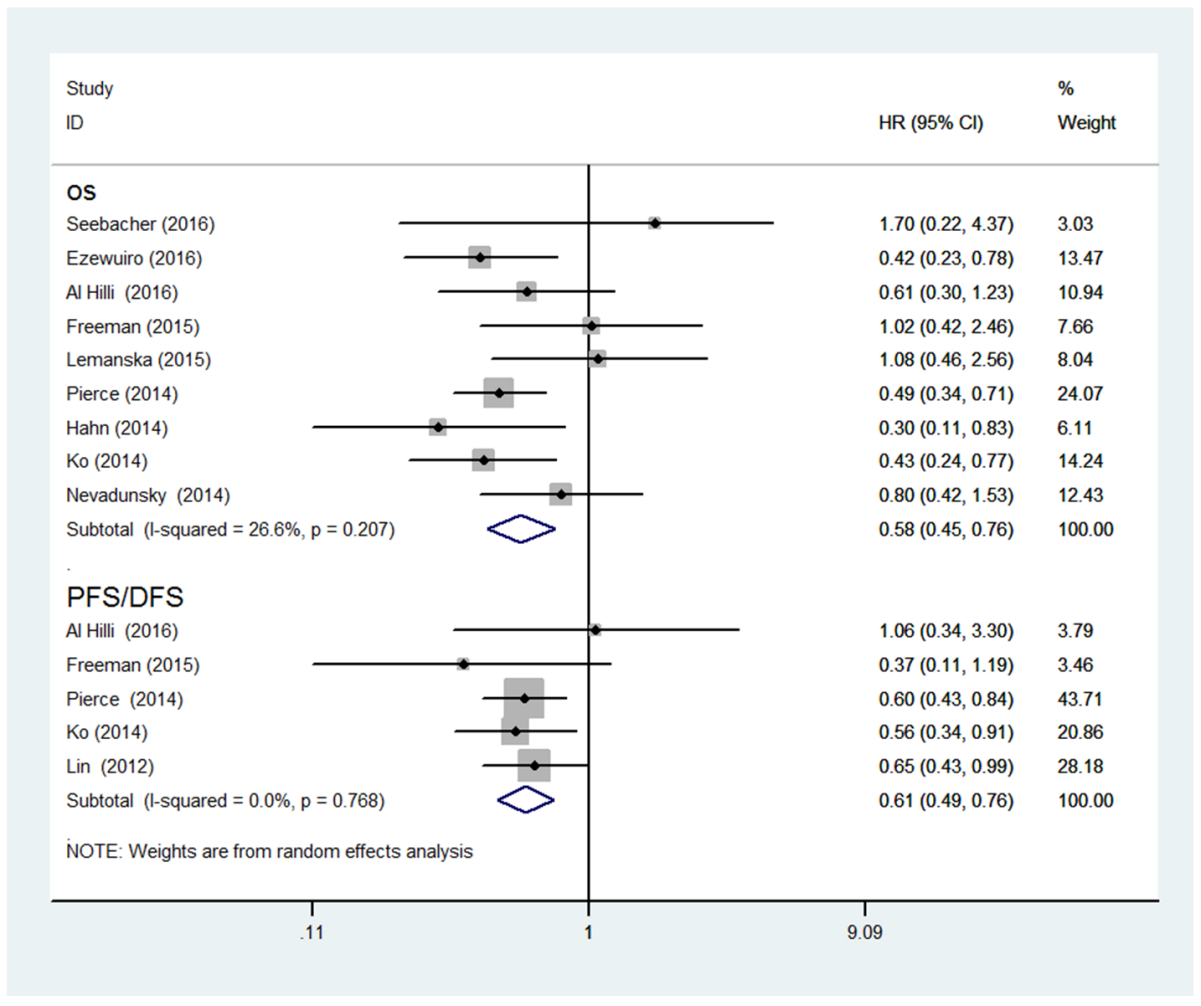
Forest plot for the association between metformin use and endometrial cancer overall survival/progression-free survival

Nine studies were included in the meta-analysis for OS. The result demonstrated that metformin users had better OS (HR, 0.58; 95% CI, 0.45 to 0.76) than non-users for endometrial cancer patients. Moderate heterogeneity was found across the studies for OS (I^2^ = 26.6%, *P* = 0.207). Associations between metformin use and endometrial cancer PFS were also evaluated. Pooled analysis showed that metformin use was associated with better PFS than non-use, with a pooled HR of 0.61 (95 % CI 0.49 to 0.76). No significant heterogeneity was noted across the studies (I^2^=0%, *P=*0.768) (Figure 2).

### Subgroup analysis of effect of metformin use on endometrial cancer OS

We investigated potential sources of inter-study heterogeneity to assess the consistency of results for OS stratified by some baseline characteristics as was shown in Table 2. For endometrial cancer, metformin users had longer OS than non-users, irrespective of study quality, number of research center, sample size or patient inclusion year, though some subgroups yielded no significant associations.

**Table 2:** Subgroup analyses in subset of included studies according to baseline characteristics for overall survival

	HR	95%CI	Heterogeneity (%)	*P*	No. of included studies
Total	0.58	0.45 to 0.76	26.6	0.207	9
Study quality					
Quality score<7	0.62	0.44 to 0.89	27.5	0.228	6
Quality score≤7	0.53	0.31 to 0.91	42.3	0.177	3
Research region					
USA	0.52	0.42 to 0.66	3.2	0.401	6
Non-USA	1.21	0.57 to 2.54	0	0.606	3
Research center					
Single	0.76	0.52 to 1.11	14.3	0.323	6
Multicenter	0.46	0.35 to 0.61	0	0.890	3
Sample size					
≥400	0.58	0.40 to 0.82	24.8	0.256	5
≥400	0.61	0.38 to 1.00	46.2	0.134	4
First inclusion year					
Before 2000	0.60	0.45 to 0.82	21.9	0.269	6
After 2000	0.52	0.27 to 1.03	53.3	0.118	3

CI= confidence intervence; DM: diabetes mellitus; HR= hazard ratios.

### Sensitivity analysis and publication bias

**Figure 3:**
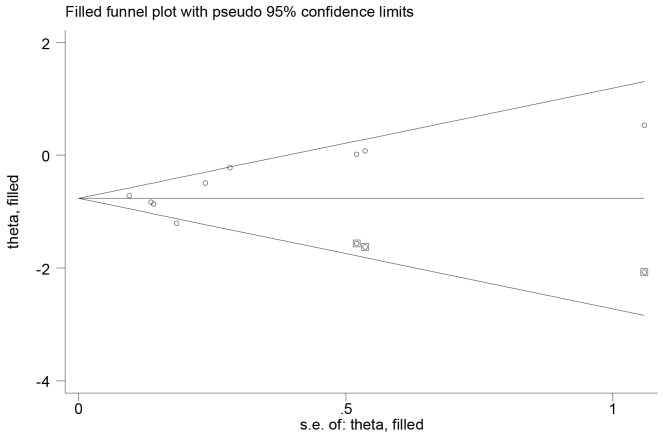
Trimmed and filled funnel plot for metformin use and endometrial cancer overall survival

**Figure 4:**
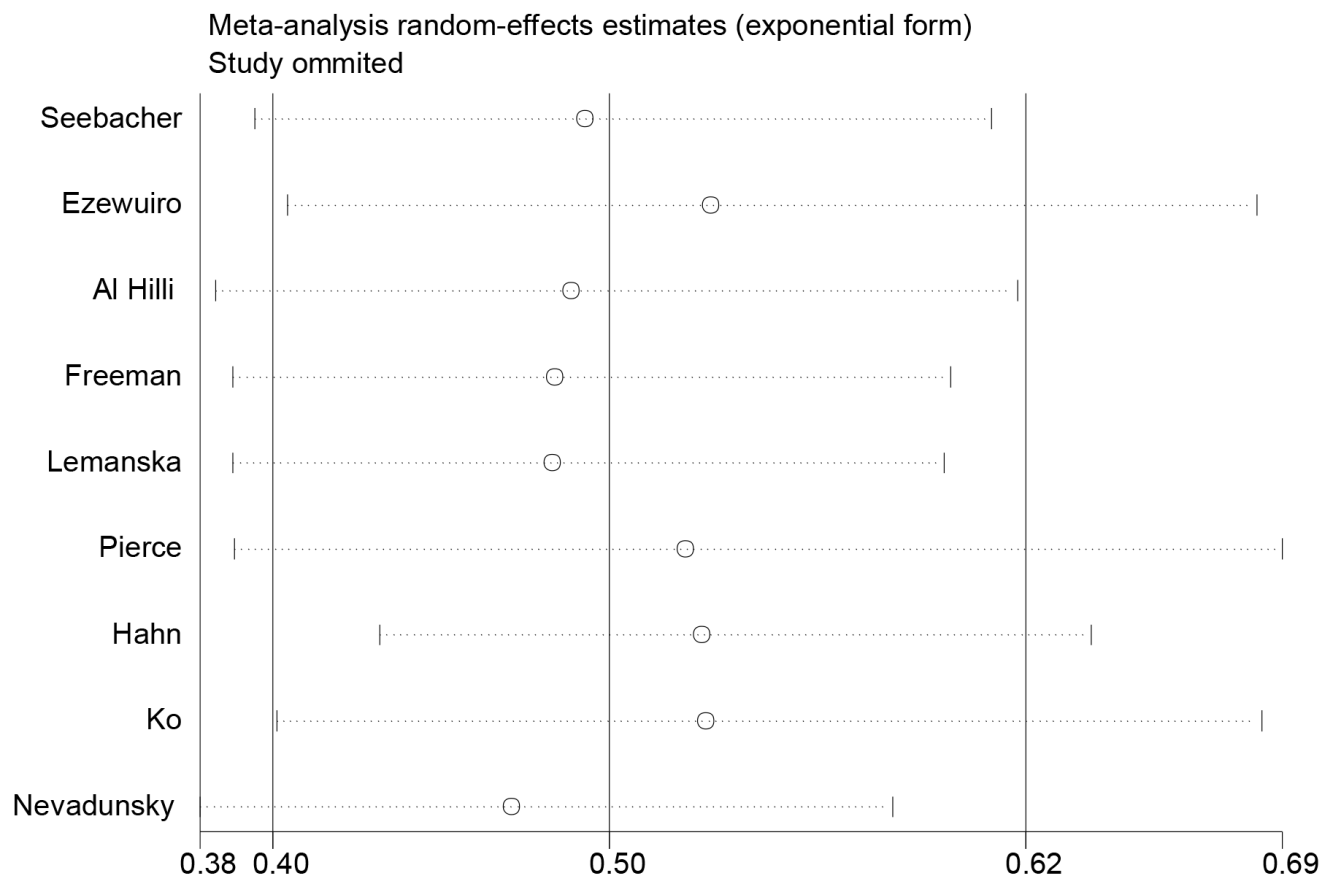
Sensitivity analysis using a random-effect model by omitting one study at a time and pooling the rest of the included studies

Sensitivity analysis was performed by excluding one study each time and recalculating the summary estimates for the other studies. We noted that the exclusions any of a specific study did not largely change the results of our primary analysis (Figure 3). We also explored the prognostic effect of metformin in patients limited to type 2 DM. The results showed that in diabetic patients with endometrial cancer, metformin use is still significantly associated with improved OS (HR, 0.54; 95% CI, 0.38 to 0.75). Egger’s test (*P*=0.155) indicated no publication bias for OS. Then we used the trim and fill method to adjust the analysis. However, our results remained unchanged (adjusted HR 0.47, 95 % CI 0.37 to 0.58) (Figure 4). We did not examine publication bias for the meta-analyses of endometrial cancer PFS due to the small study number available to make a valid statistical test.

## DISCUSSION

This is the largest and most comprehensive meta-analysis that examined the prognostic value of metformin use on the survival of endometrial cancer patients. Based on ten observational studies involving 6242 patients, of which 924 were metformin users and 5318 non-users, our results have demonstrated that metformin use is associated with an improved prognosis in terms of overall survival and progression-free survival. Compared with non-users, those who took metformin achieved an estimated 42% OS benefit and 39% PFS benefit. Furthermore, similar findings were observed using sensitivity analysis adjusted by trim and filled methods and subgroup analyses, indicating the robustness of our findings.

Metformin can reduce the overall risk of cancer incidence and cancer mortality compared with other glucose-lowering therapies for patients. Numerous clinical controlled trials and observational studies have reported such an association [33–35]. Furthermore, consistent with the findings, a substantial number of laboratory studies have implicated that metformin had antitumor properties *in vitro* and *in vivo* with various mechanisms, including reducing the circulating insulin level, promoting apoptosis, and activating metabolic pathways such as LKB1/AMP-activated protein kinase (AMPK) [36, 37], inhibiting protein synthesis by AMPK-dependent and AMPK-independent pathways [38–40], and regulating energy metabolism by modulating mircoRNA [41].

To the best of our knowledge, this is the meta-analysis with the largest sample size to systematically and quantitatively summarise the evidence from observational studies with respect to the prognostic value of metformin use in endometrial cancer. Moreover, the result is quite consistent with that of two previous published meta-analyses [42, 43] as well as the ones regarding other cancer types, such as colorectal cancer, prostate cancer and overall cancer types [44–46]. Besides the higher statistical power, we also conducted thorough subgroup analyses to test the inter-study heterogeneity.

One strength of this study is that we developed a reproducible search strategy of the major electronic databases without excluding published conference abstract to minimize publication bias. Though we observed evidence of publication bias for OS subset through funnel plot and Egger’s test, further adjusted estimates using trim and filled method did not indicate the alteration of the pooled estimates. Moreover, the subgroup analyses stratified by some baseline characteristics indicated the robustness of our primary results. Finally, a commonly used scale for prognostic studies was used to assess the methodological quality for all the included studies.

Some potential limitations must be considered when interpreting the study findings. First, the absence of individual patient data means that the present meta-analysis gives general survival estimates and does not allow the assessment of specific subgroups. It remains uncertain whether the observed metformin exposure is consistent among specific high risk patients, such as patients with advanced disease, large size or poorly differentiated tumors and we cannot exclude that the advantages of adjuvant therapy are more effective in certain subgroups of patients than the average patients in our analysis. Second, due to the limited available studies involved, heterogeneity could not thoroughly been explored, especially the lack of information of some potential confounders, especially the disease stage, the treatment duration of metformin, the treatment of the individual cancer patients and other agents, such as insulin, sulfonylureas, statins or aspirin, which had also been reported to influence the survival of cancer patients. These factors could to some extent affect the survival of endometrial cancer patients. Third, we did not search unpublished gray literature, which might miss some unpublished data with negative results. However, trim and filled method was used to test this bias and the result was in line with the primary analysis, indicating significant evidence on the association between metformin use and cancer survival. Finally, we pooled outcome measures of DFS and PFS together. Despite its similarity, this could really have brought about certain bias.

In conclusion, for patients with endometrial cancer, metformin use is associated with increased overall survival and progression-free survival. Further meta-analyses based on individual patient data are required to explore the dose-response relationship, and to further examine the nature of the association in different subgroups. Moreover, future clinical trials in endometrial cancer patients are advocated to determine whether metformin use could benefit those patients and who may benefit more from some specific therapies.

## MATERIALS AND METHODS

### Literature search and eligibility criteria

On March 6th, 2017, we performed a systematic literature search of Pubmed and Embase database for keywords related to metformin, endometrial cancer, and survival/mortality or prognosis combined with manual reference search in all selected studies based on the Preferred Reporting Items for Systematic Reviews and Meta-Analysis checklist (PRISMA). The detailed search strategies of Pubmed and Embase are provided in Supplementary Search Strategy.

Studies were considered eligible if they met the following criteria: prospective or retrospective observational cohort studies comparing prognostic information between metformin use and non-use; involving patients with the diagnosis of endometrial cancer; reporting data on mortality or progression of disease that were published in the form of full text or conference abstract; studies investigating metformin used for the treatment of diabetes instead of as adjuvant therapy for the cancer. We did not define or differentiate the detailed dose or duration of metformin use.

### Study selection and data extraction

Two reviewers (KX and YCZ) conducted the initial screening of potentially eligible literature. Further full-text record selection was performed independently by two reviewers (JFG and KX). Any disagreements were resolved by discussion or by a senior reviewer (YCZ) until consensus was reached. If multiple studies referred to the same data, we cross-checked and selected the largest or the most informative one to review. Baseline characteristics and survival data were abstracted according to the predesigned data abstraction form such as author and publication year, type of publication, research country, inclusion period, number of research center involved, study design, sample size, age, disease stage, reported endpoints, follow up period and adjusted variables.

### Study end points and quality assessment

We chose OS and DFS/PFS as our endpoints for meta-analysis. OS was defined as the time elapsing from the date of initial primary diagnosis of endometrial cancer to the date of death irrespective of the cause of death. DFS/PFS was defined as the interval between the initial primary diagnosis of endometrial cancer and the last objective follow-up information or cancer progression including cancer recurrence or metastasis. Two reviewers (KX and YC) independently evaluated the quality of the evidence for each study using a set of modified predefined criteria to assess the association between metformin use and the cancer outcomes.

### Statistical analysis

All analyses were carried out using Stata 12.0 (Stata Corporation, College Station, Tex). HR was used as an outcome measure of the prognostic value. HR < 1 indicated better survival for patients using metformin. DerSimonian and Laird random effects model was applied to calculate pooled estimates and 95% CI [47]. We chose adjusted survival estimates (HRs) reported in studies for analysis to account for confounding variables. The inter-study heterogeneity was examined by the Cochran’s Q and I^2^ statistic with an I^2^ > 50% representing significant heterogeneity [48]. We assessed the potential of publication bias by visually inspecting the funnel plot symmetry and Begg’s regression or Egger’s linear regression test [49–51]. Furthermore, sensitivity analyses were conducted by excluding one study at a time and reanalyzing the remaining ones to test whether the results changed substantially by any individual study. In addition, we used Duval’s nonparametric trim-and-fill procedure to assess the possible influence of publication bias [52]. A *P* value less than 0.05 is set to indicate statistical significance.

## SUPPLEMENTARY MATERIALS TABLES


