# Supplementary material for: Metformin and endometrial cancer survival: a quantitative synthesis of observational studies
Source: Oncotarget. 2017 Aug 2;8(39):66169–77. doi: 10.18632/oncotarget.19830 (PMC5630401; doi:10.18632/oncotarget.19830)
Supplement: Supplementary file 1 [file oncotarget-08-66169-s001.pdf]

Metformin and endometrial cancer survival: a quantitative synthesis of observational studies

SUPPLEMENTARY MATERIALS

Supplementary Search Strategy

Search included: Pubmed and Embase
Searching date: March, 2017

Search strategy for Pubmed

1. “Endometrial Neoplasms”[Mesh]
2. endometri*[Title/Abstract]
3. (Neoplasms or neoplsa* OR tumor* OR tumour* OR cancer* OR carcinoma* OR malignan*)[Title/Abstract]
4. 2 AND 3
5. 1 OR 4
6. “Metformin”[Mesh]
7. (Metformin OR Dimethylbiguanidine OR Dimethylguanylguanidine OR Glucophage OR Biguanide OR dimethyldiguanide)[Title/Abstract]
8. “Survival”[Mesh]
9. “Prognosis”[Mesh]
10. “Mortality”[Mesh]
11. (survival OR prognos* OR mortality OR predict* OR death OR fatality) [Title/Abstract]
12. 8 OR 9 OR 10 OR 11
13. 3 AND 7 AND 12

Search strategy for Embase

1. 'endometrium cancer'/exp
2. ((endometri* AND (Neoplasms or neoplsa* OR tumor* OR tumour* OR cancer* OR carcinoma* OR malignan*)) :ab,ti
3. 1 OR 2
4. 'metformin'/exp
5. (Metformin OR Dimethylbiguanidine OR Dimethylguanylguanidine OR Glucophage OR Biguanide OR dimethyldiguanide) :ab,ti
6. 4 OR 5
7. ‘mortality'/exp
8. 'survival'/exp
9. 'prognosis'/exp
10. 7 OR 8 OR 9
11. (survival OR prognos* OR mortality OR predict* OR death OR fatality) :ab,ti
12. 10 OR 11
13. 3 AND 6 AND 12

Supplementary Table 1: Selection procedure of included and excluded studies

	Reference list no.
Studies included in the meta-analysis (n = 10)	1-10
Studies excluded from meta-analysis, and reasons are listed below (n = 42)	11-52
**Reason for exclusion**	
Publication with cases included in a selected study (n =6)	11-16
No prognostic outcomes recorded (n =13)	17-29
Letters, comments, reviews or meta-analyses (n =17)	30-46
No sufficient data for analysis (n =6)	47-52

Supplementary Table 2: Methodological quality of included studies based on the Newcastle–Ottawa Scale for cohort studies

Observational studies[a]					
**Study**	**Design**	**Selection**	**Comparability**	**Outcome/exposure**	**Overall quality (max 9)**
Seebacher (2016)	Cohort	***	**	***	8
Al Hilli (2016)	Cohort	***	**	***	8
Ezewuiro (2016)	Cohort	***	**	***	8
Lemanska (2015)	Cohort	***	**	***	8
Freeman (2015)	Cohort	***	*	***	7
Hahn (2014)	Cohort	***	*	***	7
Ko (2014)	Cohort	***	**	***	8
Nevadunsky (2014)	Cohort	***	**	***	8
Pierce (2014)	Cohort	***	*	***	7
Lin (2012)	Cohort	***	*	***	7

[a] Study quality assessment of observational studies performed using the Newcastle–Ottawa scale (each asterisk represents if individual criterion within the subsection were fulfilled).

REFERENCES

1. Seebacher V, Bergmeister B, Grimm C, Koelbl H, Reinthaller A, Polterauer S. The prognostic role of metformin in patients with endometrial cancer: a retrospective study. Eur J Obstet Gynecol Reprod Biol. 2016; 203:291-296.
2. Al Hilli MM, Bakkum-Gamez JN, Mariani A, Cliby WA, Mc Gree ME, Weaver AL, Dowdy SC, Podratz KC. The effect of diabetes and metformin on clinical outcomes is negligible in risk-adjusted endometrial cancer cohorts. Gynecol Oncol. 2016; 140:270-6.
3. Freeman S. ASTRO: metformin use during RT associated with better outcomes. Oncol Rep. 2015; 11:3-4.
4. Pierce SR, Doll KM, Davidson B, Lee C, Ko EM, Snavely AC, Gehrig PA, Secord AA, Havrilesky LJ, Bae-Jump VL. Endometrial cancer outcomes in diabetic women treated with metformin, statins, and aspirin. Gynecol Oncol. 2014; 133:43.
5. Nevadunsky NS, Van Arsdale A, Strickler HD, Moadel A, Kaur G, Frimer M, Conroy E, Goldberg GL, Einstein MH. Metformin use and endometrial cancer survival. Gynecol Oncol. 2014; 132:236-240.
6. Ko EM, Walter P, Jackson A, with “Clark L, Franasiak J, Bolac C, Havrilesky LJ, Secord AA, Moore DT, Gehrig PA, Bae-Jump V. Metformin is associated with improved survival in endometrial cancer. Gynecol Oncol. 2014; 132:438-442.
7. Hahn LA, Villella JA, Chalas E, Chan KC, Jimenez EA. Metformin use is associated with improved survival in women with endometrial cancer. Gynecol Oncol 2014; 133:159.
8. Lin L, Bahng A, Rubin S, et al. Metformin use is associated with improved disease free survival in patients with endometrioid adenocarcinoma. Int J Gynecol Cancer. 2012; 22:E232.
9. Ezewuiro O, Grushko TA, Kocherginsky M, Habis M, Hurteau JA, Mills KA, Hunn J, Olopade OI, Fleming GF, Romero IL. Association of metformin use with outcomes in advanced endometrial cancer treated with chemotherapy. PLoS One. 2016; 11:e0147145.
10. Lemanska A, Zaborowski M, Spaczynski M, Nowak-Markwitz E. Do endometrial cancer patients benefit from metformin intake? Ginekol Pol. 2015; 86:419-423.
11. Al Hilli M, Bakkum-Gamez JN, Mariani A, Cliby WA, Mc Gree ME, Weaver AL, Dowdy SC, Podratz KC. The impact of diabetes and metformin on clinical outcomes is negligible in risk-adjusted endometrial cancer cohorts. Gynecol Oncol. 2015; 137:156-157.
12. Nevadunsky N, VanArsdale A, Kaur G, Frimer M, Conroy E, Einstein M, Goldberg G. Use of metformin is associated with improved endometrial cancer survival. Gynecol Oncol. 2013; 130:e74-e75.
13. Lemanska A, Zaborowski M, Magnowska M, et al. Metformin does not improve survival in endometrial cancer. Int J Gynecol Cancer. 2013; 23:335.
14. Ko EM, Walter P, Clark LH, Jackson AL, Franasiak J, Havrilesky LJ, Secord AA, Moore DT, Gehrig PA,

Bae-Jump VL. The impact of diabetes and obesity on endometrial cancer outcomes. J Clin Oncol. 2013; 31:5523
15. Ko E, Paige W, Clark L, Jackson A, Franasiak J, Havrilesky L, Alvarez-Secord A, Gehrig P, Bae-Jump V. Metformin reduces recurrence and improves survival in endometrial cancers. Gynecol Oncol. 2013; 130:e83.
16. Meichun Ko E, Franasiak J, Clark LH, Gehrig PA, Bae-Jump VL. Impact of metformin use on risk of recurrence in high-grade endometrial cancers. J Clin Oncol. 2012; 30:5009.
17. Sivalingam VN, McVey R, Gilmour K, Kitson S, Gilmour K, Ali S, Roberts C, Renehan AG, Kitchener H, Crosbie EJ. Pre-surgical window study of metformin in endometrial cancer. Int J Gynecol Cancer. 2014; 24:1593.
18. Shao R, Li X, Feng Y, Lin JF, Billig H. Direct effects of metformin in the endometrium: a hypothetical mechanism for the treatment of women with PCOS and endometrial carcinoma. J Exp Clin Cancer Res. 2014; 33:41
19. Perez-Lopez FR. Metformin treatment and evolution of endometrial cancer. Climacteric. 2014; 17:207-209.
20. Mitsuhashi A, Usui H, Satoh Y, Kiyokawa T, Shozu M. A phase II study of medroxyprogesterone acetate plus metformin as fertility-sparing treatment for atypical endometrial hyperplasia and endometrial cancer. J Clin Oncol. 2014; 32:5592.
21. Hawkes AL, Quinn M, Gebski V, Armes J, Brennan D, Janda M; feMME Trial Committee, Obermair A. Improving treatment for obese women with early stage cancer of the uterus: Rationale and design of the levonorgestrel intrauterine device(plus or minus)Metformin(plus or minus) weight loss in endometrial cancer (feMME) trial. Contemp Clin Trials. 2014; 39:14-21.
22. Bershteyn LM, Vasil'ev DA, Ievleva AG, Imyanitov EN. Hormonal, metabolic and genetic predictors of metformin efficacy in patients with diabetes mellitus and cancer. Diab Mellitus. 2014; 21-28.
23. Umene K, Banno K, Kisu I, Yanokura M, Nogami Y, Tsuji K, Masuda K, Ueki A, Kobayashi Y, Yamagami W, Tominaga E, Susumu N, Aoki D. New candidate therapeutic agents for endometrial cancer: potential for clinical practice. Oncol Rep. 2013; 29:855-860.
24. Soliman P, Broaddus R, Westin S, Iglesias D, Munsell M, Schmandt R, Celestino J, Burzawa J, Lu K. Phase 0 study: prospective evaluation of the molecular effects of metformin on the endometrium in women with newly diagnosed endometrial cancer. Gynecol Oncol. 2013; 130:e13.
25. Schuler KM, Rambally BS, DiFurio MJ, Gehrig PA, Bae-Jump VL. A preoperative window study of metformin for the treatment of endometrial cancer. J Clin Oncol. 2013; 31:5519.
26. Myers AP. New strategies in endometrial cancer: targeting the PI3K/mTOR pathway—the devil is in the details. Clin Cancer Res. 2013; 19:5264-5274.
27. Soffer D, Shi J, Chung J, et al. Does metformin use among women with type II diabetes reduce the risk of gynecologic cancers? Int J Gynecol Cancer. 2012; 22:E162.
28. Martin-Castillo B, Vazquez-Martin A, Oliveras-Ferraros C, Menendez JA. Metformin and cancer: Doses, mechanisms and the dandelion and hormetic phenomena. Cell Cycle. 2010; 9:1057-1064.
29. Bae-Jump VL, Kaufman D, Gehrig PA. Metformin as a novel chemotherapeutic strategy for endometrial cancer. Clin Transl Sci. 2010; 3:S35.
30. Klil-Drori AJ, Azoulay L, Pollak MN. Cancer, obesity, diabetes, and antidiabetic drugs: is the fog clearing? Nat Rev Clin Oncol. 2017; 14:85-99.
31. Irie H, Banno K, Yanokura M, Iida M, Adachi M, Nakamura K, Umene K, Nogami Y, Masuda K, Kobayashi Y, Tominaga E, Aoki D. Metformin: a candidate for the treatment of gynecological tumors based on drug repositioning. Oncol Lett. 2016; 11:1287-1293.
32. Chae YK, Arya A, Malecek MK, Shin D, Carneiro B, Chandra S, Kaplan J, Kalyan A, Altman JK, Platanias L, Giles F. Repurposing metformin for cancer treatment: current clinical studies. Oncotarget. 2016; 7:40767-40780. http://dx.doi.org/10.18632/oncotarget.8194.
33. Provinciali N, Lazzeroni M, Cazzaniga M, Gorlero F, Dunn BK, DeCensi A. Metformin: risk-benefit profile with a focus on cancer. Expert Opin Drug Saf. 2015; 14:1573-1585.
34. Morales DR, Morris AD. Metformin in cancer treatment and prevention. Annu Rev Med. 2015; 66:17-29.
35. Imai A, Ichigo S, Matsunami K, Takagi H, Yasuda K. Clinical benefits of metformin in gynecologic oncology. Oncol Lett. 2015; 10:577-582.
36. Febbraro T, Lengyel E, Romero IL. Old drug, new trick: repurposing metformin for gynecologic cancers? Obstet Gynecol Surv. 2015; 70:91-92.
37. Zhang ZJ, Li S. The prognostic value of metformin for cancer patients with concurrent diabetes: a systematic review and meta-analysis. Diabetes Obes Metab. 2014; 16:707-710.
38. Stine JE, Bae-Jump V. Metformin and gynecologic cancers. Obstet Gynecol Surv. 2014; 69:477-489.
39. Wang T, Ning G, Bloomgarden Z. Diabetes and cancer relationships. J Diabetes. 2013; 5:378-390.
40. Pulito C, Sanli T, Rana P, Muti P, Blandino G, Strano S. Metformin: On ongoing journey across diabetes, cancer therapy and prevention. Metabolites. 2013; 3:1051-1075.
41. Hajjar J, Habra MA, Naing A. Metformin: an old drug with new potential. Expert Opin Investig Drugs. 2013; 22:1511-1517.

42. Franciosi M, Lucisano G, Lapice E, Strippoli GF, Pellegrini F, Nicolucci A. Metformin therapy and risk of cancer in patients with type 2 diabetes: systematic review. PLoS One. 2013; 8:e71583.
43. Kourelis TV, Siegel RD. Metformin and cancer: new applications for an old drug. Med Oncol. 2012; 29:1314-1327.
44. Schmandt RE, Iglesias DA, Co NN, Lu KH. Understanding obesity and endometrial cancer risk: opportunities for prevention. Am J Obstet Gynecol. 2011; 205:518-525.
45. Belda-Iniesta C, Pernia O, Simo R. Metformin: a new option in cancer treatment. Clin Transl Oncol. 2011; 13:363-367.
46. Papanas N, Maltezos E, Mikhailidis DP. Metformin and cancer: licence to heal? Expert Opin Investig Drugs. 2010; 19:913-917.
47. Hall C, Stone RL, Gehlot A, Zorn KK, Burnett AF. Use of metformin in obese women with type I endometrial cancer is associated with a reduced incidence of cancer recurrence. Int J Gynecol Cancer. 2016; 26:313-317.
48. Ko EM, Sturmer T, Hong JL, Castillo WC, Bae-Jump V, Funk MJ. Metformin and the risk of endometrial cancer: a population-based cohort study. Gynecol Oncol. 2014; 133:32.
49. Hawkes AL, Quinn M, Gebski V, Armes J, Brennan D, Janda M; feMME Trial Committee, Obermair A. Improving treatment for obese women with early stage cancer of the uterus: rationale and design of the levonorgestrel intrauterine device +/- metformin +/- weight loss in endometrial cancer (feMME) trial. Contemp Clin Trials. 2014; 39:14-21.
50. Ezewuiro OC, Habis MI, Kocherginsky M, Hunn J, Olopade OI, Grushko TA, Romero IL, Fleming GF. A retrospective analysis of the relationship between diabetes, metformin use, and survival in advanced endometrial cancer patients treated with chemotherapy. J Clin Oncol. 2014; 32.
51. Hall C, Stone R, Burnett A. Metformin may reduce the incidence of endometrial cancer recurrence in obese women. Int J Gynecol Cancer. 2013; 23:1066.
52. Woods C, Radivoyevitch T, Manochakian R, et al. Impact of metformin use in diabetic women receiving endometrial cancer treatment. Int J Gynecol Cancer. 2012; 22:E1155.
